# Pancolitis Associated With COVID-19 Infection: A Case Report

**DOI:** 10.7759/cureus.20307

**Published:** 2021-12-09

**Authors:** Siham Hussien, Bereket K Tewoldemedhin, Dena H Tran, Aseem Sood, Miriam B Micheal

**Affiliations:** 1 Internal Medicine, University of Maryland Midtown Campus, Baltimore, USA; 2 Internal Medicine, Suburban Community Hospital (Lower Bucks Hospital), Bristol, USA

**Keywords:** bloody diarrhea, gastrointestinal, pancolitis, sars-cov-2, covid-19

## Abstract

Coronavirus disease 2019 (COVID-19) is caused by the severe acute respiratory syndrome coronavirus 2 (SARS-CoV-2). COVID-19 infection commonly affects the pulmonary system, ranging from being asymptomatic to having mild upper respiratory tract infection symptoms, to having severe cases causing multi-organ failure. However, COVID-19 infection involving the gastrointestinal (GI) tract leading to pancolitis is an extremely rare complication. We present a rare case of a patient who presented with pancolitis and on testing for admission found to be positive for COVID-19. We will explore the GI tropism and the mechanism of COVID-19 infection with gastrointestinal symptoms of pancolitis.

## Introduction

Gastrointestinal (GI) symptoms are reported in approximately 35% of patients with COVID-19 infections, with one reported case of pancolitis [1,2]. Pancolitis is a severe form of inflammation of the colon, affecting the entire colon. We present a case of a 20-year-old male with no history of inflammatory bowel disease who presented with bloody diarrhea, as well as flu-like symptoms, and tested positive by rapid antigen testing for coronavirus disease 2019 (COVID-19) infection.

We will discuss the different ways in which the virus causes GI symptoms, with the first mechanism being through the binding of angiotensin-converting enzyme 2 (ACE2) receptors that are found in the GI tract as well as alveolar cells in the lungs and another being through pro-inflammatory cytokine (PIC)-mediated damage [3].

## Case presentation

A previously healthy 20-year-old male with a history of autism spectrum disorder and attention deficit hyperactivity disorder presented to the emergency department (ED) with complaints of bloody stool for two days. The patient presented to the ED with flu-like symptoms including fever, chills, malaise, back pain, and watery diarrhea. The patient did not exhibit pulmonary symptoms such as cough or shortness of breath. He subsequently tested positive for COVID-19 infection. He was managed with supportive care and was discharged home with quarantine instructions on the second day of hospitalization. He returned to the ED two days later reporting 5-10 episodes of maroon-colored loose stool associated with intermittent periumbilical pain that was dull in character and relieved with defecation. He reported anorexia and nausea. He did not have similar symptoms in the past. The patient denied a history of easy bruising, use of nonsteroidal anti-inflammatory drugs (NSAIDs) or anticoagulation, recent travel, and ingestion of raw, undercooked meals. He does not have a history of abdominal surgery and does not take any medications including over-the-counter medications, herbs, or supplements. He denied a family history of gastrointestinal illness, malignancy, and inflammatory bowel disease. The patient does not smoke cigarettes, drink alcohol, or use other illicit drugs.

**Figure 1:**
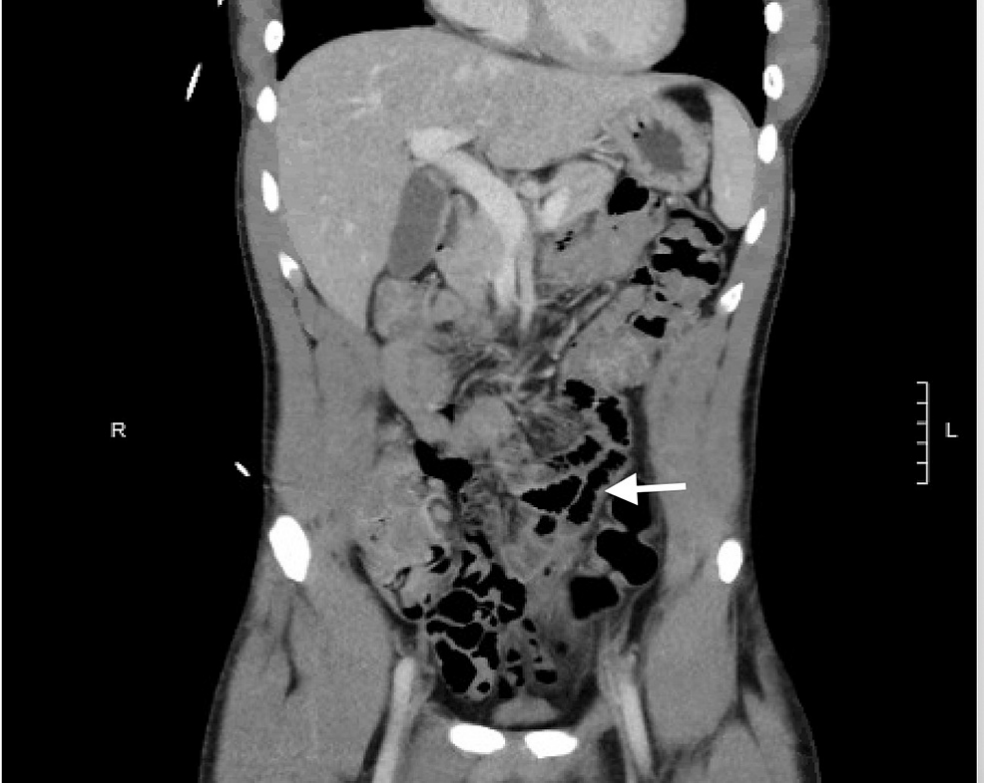
CT scan showing reactive small mesenteric lymphadenopathy and circumferential wall thickening of the colon with liquid stool noted in the rectum minimal surrounding fat stranding suggestive of pancolitis.

His vital signs were as follows: temperature, 37°C; heart rate, 106 beats/minute; blood pressure, 116/60 mmHg; respiratory rate, 18 breaths/minute; and oxygen saturation, 97% on room air. Physical examination revealed a soft, non-distended abdomen with normal bowel sounds, and no abdominal tenderness was noted on palpation. In addition, there were no anal fissures or hemorrhoids noted on digital rectal examination. The laboratory results showed white blood cell of 4.1 K/mcL (normal range: 3.1-10.5 K/mcL), hemoglobin of 15 g/dL (normal range: 12.6-17.4 g/dL), mean corpuscular volume of 81.4 fL (normal range: 80-96 fL), mean corpuscular hemoglobin of 26.9 pg (normal range: 28-33 pg), and platelets of 154 K/mcL (normal range: 153-367 K/mcL). The automated differential showed absolute lymphopenia of 1.1 K/mcL (normal range: 1.3-3.5 K/mcL). His protime was 13.4 seconds (normal range: 12.1-14.9 seconds), International Normalized Ratio (INR) was 1.01, and activated prothrombin time was 30.5 seconds (normal range: 23.9-41.3 seconds). The inflammatory marker C-reactive protein was elevated at 6.80 mg/dL (normal range: 0-0.99 mg/dL). Severe acute respiratory syndrome coronavirus 2 (SARS-CoV-2) (COVID-19) RNA was positive. Computed tomography (CT) of the abdomen and pelvis showed moderate proximal and mild distal circumferential wall thickening of the colon with liquid stool noted in the rectum minimal surrounding fat stranding suggestive of pancolitis likely infectious/inflammatory in etiology with reactive mesenteric lymphadenopathy (Figure 1). Furthermore, there were no inflammatory changes in the pericecal area to suggest appendicitis.

On the second day of admission, the symptoms spontaneously resolved; he had overall improvement of his abdominal pain and nausea and did not have any further bloody bowel movements. Antibiotics were not administered, and no further workup, including colonoscopy, was warranted given that all symptoms had resolved. Follow-up with the patient after discharge confirmed complete resolution of symptoms.

## Discussion

With the evolution of the COVID-19 pandemic, there has been emerging data regarding the extrapulmonary manifestations of COVID-19 infection. Digestive symptoms are a common presentation of COVID-19 infection, in a cross-sectional study done across multiple facilities, which demonstrated that patients can present with isolated GI symptoms [3]. Pancolitis is typically associated with ulcerative colitis, and the association in some reports shows that the frequency can be as high as 20%-40%. In several studies, inflammatory bowel disease was not found to increase the incidence or severity of COVID-19 [4-7].

Our patient did not have a prior history of inflammatory bowel disease and remains disease-free on follow-up in the GI clinic several months later. This makes the presentation with an acute pancolitis attributable to the simultaneous positive COVID-19 infection. The other differential diagnosis for acute onset bloody diarrhea includes infectious causes such as bacterial *Escherichia coli* 0157:H7, *Salmonella*, *Shigella*, viral infections, and parasitic infections. Other causes in this differential include diverticulosis and arteriovenous malformations. However, given the unrevealing history and laboratory studies, the cause is most likely a presentation of COVID-19 infection. Limited data have suggested that diarrheal symptoms in patients with COVID-19 have been associated with a favorable prognosis and with the detection of the virus RNA in stool [8-10].

There are several possible ways by which the virus can cause damage to the GI tract and cause symptoms. The first one is by binding to the human angiotensin-converting enzyme 2 (ACE2) receptor, and the second is by causing local inflammatory response [3]. It has been shown that the same ACE2 RNA receptors that are expressed in type II alveolar cells of the lung are also expressed in the gastrointestinal tract [7,8]. The ACE2 receptors allow for viral replication and transmission, along with possible upregulation of pro-inflammatory cytokines in order to fight the virus [7,9]. In another study that examined the expression of pro-inflammatory cytokines (PICs) in cells that are positive for ACE2 and SARS-CoV S proteins, the data suggest that PICs are overproduced in the SARS-CoV-infected ACE2 cells [8,11,12]. The overproduction of pro-inflammatory cytokines causes damage to both infected and uninfected cells in the lungs and other organs, which include the cells of the gastrointestinal tract [9,11].

## Conclusions

Pancolitis should be considered as a manifestation of acute COVID-19 infection. There have been cases of exacerbation in patients with pre-existing inflammatory bowel disease. However, it is rarely reported to present in patients without a prior history. It is important to recognize extrapulmonary manifestations of COVID-19, such as the gastrointestinal symptoms of this patient, in order to effectively diagnose and treat the disease that may otherwise be misdiagnosed as an unrelated condition.

## References

[1] Nobel YR, Phipps M, Zucker J, Lebwohl B, Wang TC, Sobieszczyk ME, Freedberg DE (2020). Gastrointestinal symptoms and coronavirus disease 2019: a case-control study from the United States. Gastroenterology.

[2] Asil RS, Mahmoodi S, Zamani A, Hakakzadeh A, Jamali E (2021). Abdominal pain and diffuse colitis following COVID-19 infection: report of a case. Int J Surg Case Rep.

[3] Pan L, Mu M, Yang P (2020). Clinical characteristics of COVID-19 patients with digestive symptoms in Hubei, China: a descriptive, cross-sectional, multicenter study. Am J Gastroenterol.

[4] Mao R, Liang J, Shen J (2020). Implications of COVID-19 for patients with pre-existing digestive diseases. Lancet Gastroenterol Hepatol.

[5] Norsa L, Indriolo A, Sansotta N, Cosimo P, Greco S, D'Antiga L (2020). Uneventful course in patients with inflammatory bowel disease during the severe acute respiratory syndrome coronavirus 2 outbreak in Northern Italy. Gastroenterology.

[6] Rubin DT, Feuerstein JD, Wang AY, Cohen RD (2020). AGA clinical practice update on management of inflammatory bowel disease during the COVID-19 pandemic: expert commentary. Gastroenterology.

[7] Zhao Zhao, Zhao Z, Wang Y, Zhou Y, Ma Y, Zuo W (2020). Single-cell RNA expression profiling of ACE2, the putative receptor of Wuhan 2019-nCov. bioRxiv.

[8] He L, Ding Y, Zhang Q (2006). Expression of elevated levels of pro-inflammatory cytokines in SARS-CoV-infected ACE2+ cells in SARS patients: relation to the acute lung injury and pathogenesis of SARS. J Pathol.

[9] von Grotthuss M, Wyrwicz LS, Rychlewski L (2003). mRNA cap-1 methyltransferase in the SARS genome. Cell.

[10] Rota PA, Oberste MS, Monroe SS (2003). Characterization of a novel coronavirus associated with severe acute respiratory syndrome. Science.

[11] Cheung KS, Hung IF, Chan PP (2020). Gastrointestinal manifestations of SARS-CoV-2 infection and virus load in fecal samples from a Hong Kong cohort: systematic review and meta-analysis. Gastroenterology.

[12] Luo S, Zhang X, Xu H (2020). Don’t overlook digestive symptoms in patients with 2019 novel coronavirus disease (COVID-19). Clin Gastroenterol Hepatol.

